# Health and economic burden of influenza‐associated illness in South Africa, 2013‐2015

**DOI:** 10.1111/irv.12650

**Published:** 2019-06-11

**Authors:** Stefano Tempia, Jocelyn Moyes, Adam L. Cohen, Sibongile Walaza, Ijeoma Edoka, Meredith L. McMorrow, Florette K. Treurnicht, Orienka Hellferscee, Nicole Wolter, Anne von Gottberg, Athermon Nguweneza, Johanna M. McAnerney, Halima Dawood, Ebrahim Variava, Cheryl Cohen

**Affiliations:** ^1^ Influenza Division Centers for Disease Control and Prevention Atlanta Georgia; ^2^ Influenza Program Centers for Disease Control and Prevention Pretoria South Africa; ^3^ Centre for Respiratory Diseases and Meningitis National Institute for Communicable Diseases of the National Health Laboratory Service Johannesburg South Africa; ^4^ MassGenics Duluth Georgia; ^5^ Faculty of Health Sciences, School of Public Health University of the Witwatersrand Johannesburg South Africa; ^6^ Expanded Programme on Immunization, Department of Immunization, Vaccines and Biological World Health Organization Geneva Switzerland; ^7^ Priority Cost Effectiveness Lessons for System Strengthening South Africa, South Africa Medical Research Council, Wits Center for Health Economic and Decision Science, School of Public Health University of the Witwatersrand Johannesburg South Africa; ^8^ Faculty of Health Sciences, School of Pathology University of the Witwatersrand Johannesburg South Africa; ^9^ Department of Medicine Greys Hospital Pietermaritzburg South Africa; ^10^ Caprisa University of KwaZulu‐Natal Pietermaritzburg South Africa; ^11^ Department of Medicine Klerksdorp‐Tshepong Hospital Complex Klerksdorp South Africa; ^12^ Faculty of Health Sciences, Department of Medicine University of the Witwatersrand Johannesburg South Africa; ^13^ Perinatal HIV Research Unit University of the Witwatersrand Johannesburg South Africa

**Keywords:** economic burden, influenza, South Africa, years of life lost

## Abstract

**Background:**

Economic burden estimates are essential to guide policy‐making for influenza vaccination, especially in resource‐limited settings.

**Methods:**

We estimated the cost, absenteeism, and years of life lost (YLL) of medically and non‐medically attended influenza‐associated mild and severe respiratory, circulatory and non‐respiratory/non‐circulatory illness in South Africa during 2013‐2015 using a modified version of the World Health Organization (WHO) worksheet based tool for estimating the economic burden of seasonal influenza. Additionally, we restricted the analysis to influenza‐associated severe acute respiratory illness (SARI) and influenza‐like illness (ILI; subsets of all‐respiratory illnesses) as suggested in the WHO manual.

**Results:**

The estimated mean annual cost of influenza‐associated illness was $270.5 million, of which $111.3 million (41%) were government‐incurred costs, 40.7 million (15%) were out‐of‐pocket expenses, and $118.4 million (44%) were indirect costs. The cost of influenza‐associated medically attended mild illness ($107.9 million) was 2.3 times higher than that of severe illness ($47.1 million). Influenza‐associated respiratory illness costs ($251.4 million) accounted for 93% of the total cost. Estimated absenteeism and YLL were 13.2 million days and 304 867 years, respectively. Among patients with influenza‐associated WHO‐defined ILI or SARI, the costs ($95.3 million), absenteeism (4.5 million days), and YLL (65 697) were 35%, 34%, and 21% of the total economic and health burden of influenza.

**Conclusion:**

The economic burden of influenza‐associated illness was substantial from both a government and a societal perspective. Models that limit estimates to those obtained from patients with WHO‐defined ILI or SARI substantially underestimated the total economic and health burden of influenza‐associated illness.

## 1 INTRODUCTION

Influenza is a vaccine‐preventable infectious disease that causes substantial morbidity and mortality globally every year, in particular among young and elderly individuals and persons with underlying medical conditions.^1^, ^2^, ^3^, ^4^ Global studies have suggested a higher burden of influenza‐associated severe illness, including death, in Africa compared to other regions.^1^, ^2^ Nonetheless, influenza vaccine use remains low on the continent.^5^ Disease and economic burden estimates are essential to guide policy‐making on influenza vaccination, especially in resource‐limited settings; however, the disease and economic burden of influenza‐associated illness remain poorly understood in Africa.

In South Africa, a middle‐income country of 54.8 million people in 2015, over 10 million mild, 128 000 severe‐non‐fatal and 11 000 fatal influenza‐associated illness episodes are estimated to occur annually with a heavy burden among young and old individuals and persons with chronic medical conditions, including HIV and tuberculosis infection.^3^, ^4^, ^6^, ^7^, ^8^, ^9^, ^10^, ^11^ Approximately one million doses of influenza vaccine are available in the private sector annually, and approximately the same number of doses is available annually in the public sector since 2010. Vaccination guidelines are revised annually, and influenza vaccination is recommended for groups at increased risk of influenza‐associated severe illness with the highest priority given, in recent years, to pregnant women and HIV‐infected individuals.^12^ In addition, an influenza policy that also provides the implementation framework for influenza immunization was adopted in 2017.^13^ Nonetheless, the number of doses of influenza vaccine remains insufficient to cover the recommended risk groups, estimated to be over 20 million individuals.^13^

In South Africa, like in many other countries, primary drivers of vaccine policy are the cost of the vaccine, its delivery, and the economic impact of the disease. South Africa has a policy of introducing new vaccines or increasing vaccination coverage only after careful consideration of the evidence of disease burden and the potential costs and benefits from such vaccines. However, economic burden estimates of influenza‐associated illness in South Africa are lacking. In this study, we aimed to estimate the mean annual direct and indirect costs, absenteeism, and years of life lost (YLL) from influenza‐associated illness in South Africa from 2013 through 2015.

## 2 METHODS

### 2.1 Definitions

We estimated the economic burden from a societal perspective. Costs in South African Rands were converted to 2015 US dollar using average monthly South African Rand to US dollar exchange rate in 2015. All costs were expressed in 2015 prices using the South Africa all‐items Consumer Price Index.^14^

We aggregated influenza‐associated illness within four syndromic endpoints according to published literature from South Africa,^6^ namely: (a) acute respiratory illness (ie, those meeting the World Health Organization [WHO] severe acute respiratory illness [SARI] and influenza‐like illness [ILI] case definitions, which are a subset of all‐respiratory illness)^15^; (b) all‐respiratory illness; (c) all‐circulatory illness; and (d) all‐medical non‐respiratory/non‐circulatory illness. The main analysis focused on syndromes (b–d), and the total economic burden was obtained by summing the cost among these three syndromes, whereas syndrome (a) was used as sensitivity analysis (ie, minimum estimate). We considered healthcare attendance of influenza‐associated illness in two categories: (a) medically attended: attended by a registered medical care provider/institution excluding pharmacies and (b) non‐medically attended: not attended by a registered medical care provider/institution including pharmacies and traditional healers. We defined the severity of the abovementioned influenza‐associated illnesses in three levels as follows: (a) mild: medically and non‐medically attended illness not warranting hospitalization; (b) severe: medically and non‐medically attended illness warranting hospitalization, including deaths; and (c) in‐ and out‐of‐hospital deaths.

We considered the cost of influenza‐associated illness in three categories as follows: (a) direct medical costs: costs related to treatment incurred both within and outside health facilities (ie, costs of ambulatory care, hospitalization, pharmaceuticals, and consultation with traditional healers); (b) direct non‐medical costs: illness‐related expenditures that do not relate directly to medical treatment (eg, transportation costs to hospital/clinics, additional food costs, and extra expenses for accommodation for both patient and caregiver); and (c) indirect costs: the value of lost productivity because of reduced working time (for both patient and caregiver) during the illness episode or while receiving care. Medical and medication costs not covered by healthcare facilities, consultation with traditional healers, and direct non‐medical costs were considered out‐of‐pocket expenditures.

### 2.2 Data sources

For estimation of the economic burden and YLL from influenza‐associated illness, we used a combination of published or publically available data^6^, ^16^, ^17^, ^18^, ^19^, ^20^, ^21^, ^22^, ^23^, ^24^, ^25^, ^26^, ^27^, ^28^, ^29^, ^30^, ^31^ as well as data collected through laboratory‐confirmed influenza surveillance and costing studies. The list of the data sources and their use in the estimation approach are provided in Table 1, whereas the detailed description of each data source is provided as Appendix S1. All estimates of influenza disease burden, either for medically or non‐medically attended illness, were based on pre‐existing mean annual national estimates for 2013‐2015 (DS2 in Table 1 and Appendix S1).^6^

**Table 1:** Data sources used for the estimation of the economic burden of and years of life lost for influenza‐associated illness in South Africa, 2013‐2015

Data source (DS) number	Data source and description	Input/use in the estimation approach	Ref.
DS1	Projections of the 2011 South Africa census for 2013‐2015.	Population denominators.	14, 15, 16
DS2	National estimates of influenza‐associated illness during 2013‐2015.	Rates of influenza‐associated illness across syndromes (respiratory, circulatory, and non‐respiratory/non‐circulatory), levels of severity (mild, severe and fatal), and healthcare attendance (medically and non‐medically attended).	6
DS3	Laboratory‐confirmed influenza surveillance data among inpatients with SARI and outpatients with ILI at selected surveillance sites during 2013‐2015.	Length of hospitalization and proportion of in‐hospital procedures (ie, admission to ICU, chest X‐rays, oxygen therapy, medications, and laboratory tests) among influenza‐positive patients with SARI. Proportion of medications among influenza‐positive patients with ILI.	Primary data collection
DS4	Hospitalization data from a large private hospital network (NetCare) active in 7/9 provinces.	Length of hospitalization among patients hospitalized with circulatory or non‐respiratory/non‐circulatory syndromes.	17
DS5	Cost of hospitalization and outpatient consultation.	Itemized unit cost (ie, facility fee, consultation, ICU, chest X‐ray, oxygen therapy, medications, and laboratory tests) for hospitalization and outpatient consultation. Cost per patient day equivalent for hospitalization and average outpatient consultation cost.	18, 19, 20, 21 22, 23
DS6	Costing study of laboratory‐confirmed influenza inpatients with SARI and outpatients with ILI at selected surveillance sites during 2014.	Direct medical and non‐medical costs incurred by the patient/caregiver and absenteeism for medically attended illness (out‐of‐pocket expenditures).	Primary data collection
DS7	Community costing survey implemented in 4/9 provinces of South Africa.	Direct medical and non‐medical costs incurred by the patient/caregiver and absenteeism for non‐medically attended illness (out‐of‐pocket expenditures).	24
DS8	Healthcare utilization survey among individuals with SARI or ILI in three South African communities.	Proportion of non‐medically attended illness that sought care with pharmacies or traditional healers.	25, 26
DS9	Minimum wages and unemployment rate.	Indirect cost due to loss of productivity.	27, 28
DS10	Life expectancy for South Africa during 2013‐2015.	Years of life lost.	29

Abbreviations: ICU, intensive care unit; ILI, influenza‐like illness; SARI, severe acute respiratory illness.

### 2.3 Estimation of the economic burden of influenza‐associated illness

To estimate the national economic burden of influenza‐associated illness, we used the WHO manual and toolkit, a worksheet based tool, for estimating the economic burden of seasonal influenza^32^ with the following modifications: (a) we included direct and indirect cost estimates for patients and caregivers for non‐medically attended severe illness, as a high proportion (56.1%) of influenza‐associated severe illness is not medically attended in South Africa,^6^ and (b) we included indirect costs for patients and caregivers for non‐medically attended mild illness, as a high proportion (74.4%) of influenza‐associated mild illness is not medically attended in South Africa.^6^ Furthermore, of these non‐medically attended illness episodes, a high proportion (57.3%) results in absenteeism from school or work for the patient and/or the caregiver.^26^ For each data input in the WHO toolkit, we inputted the main estimate as well as the 95% CI as required.^32^

We reported total costs summed across the three main endpoints as well as cost estimates disaggregated by the three main syndromic endpoints considered in this study (ie, all‐respiratory, all‐circulatory, and non‐respiratory/non‐circulatory). Estimates were parametrized and generated within six age categories (ie, <1, 1‐4, 5‐19, 20‐44, 45‐64, and ≥65 years of age) and subsequently aggregated.

In addition, we implemented a sensitivity analysis using the WHO toolkit without the abovementioned modifications and restricted to influenza‐associated ILI and SARI (a subset of all‐respiratory illness) as suggested in the WHO manual.^32^ We also reported the economic burden of influenza‐associated illness per capita and as a proportion of the gross domestic product (GDP) for comparison with other studies globally. South Africa's mean annual GDP during 2013‐2015 was $344.9 billion.^33^

#### 2.3.1 Direct cost estimation

In the main analysis for medically attended severe illness, data on quantities of resources used during hospitalization were obtained from influenza‐positive patients hospitalized with SARI (ie, number of admissions to ICU, chest X‐rays, oxygen therapy, medications, and laboratory tests—DS3 in Table 1 and Appendix S1) through routine influenza surveillance conducted in seven public hospitals across the country. Unit costs of hospitalization/procedures, medications, and laboratory testing were obtained from the uniform patient fee schedule of the National Department of Health and the state price list of the National Health Laboratory Service (DS5 in Table 1 and Appendix S1). Unit cost was applied to average quantity of resources used per influenza‐associated SARI episode to estimate ancillary costs (ie, costs of procedures, medications, and laboratory tests). Routine service costs per day (eg, facility and consultation fees) were multiplied by the mean length of hospitalization, which was estimated within syndromes using DS3 and DS4 in Table 1 and Appendix S1. Routine service costs and ancillary costs were summed to obtain the mean total cost of influenza‐associated SARI hospitalization per episode. This figure was multiplied by national estimates of the number of influenza‐associated severe illness episodes (obtained from DS1 and DS2 in Table 1 and Appendix S1) across the syndromes evaluated in this study (ie, acute‐respiratory, all‐respiratory, all‐circulatory, and non‐respiratory/non‐circulatory). This was done because hospitalization costs among influenza‐associated non‐SARI illness were not available. In addition, we obtained the mean out‐of‐pocket expenditure (for patient and caregiver) per influenza‐associated SARI episode using data from a costing study (DS6 in Table 1 and Appendix S1) conducted at the same hospitals where SARI surveillance was implemented. A similar approach was used to estimate the mean consultation cost and out‐of‐pocket expenses per episode of influenza‐associated ILI and multiplied by national estimates of the number of influenza‐associated mild illness episodes (using DS1 and DS2 in Table 1 and Appendix S1) across the syndromes evaluated in this study.

For non‐medically attended mild and severe illness, the quantity of resources used was obtained from community costing surveys (DS7 in Table 1 and Appendix S1) and healthcare utilization surveys (DS8 in Table 1 and Appendix S1). The estimated costs per episode were multiplied by national estimates of the number of non‐medically attended influenza‐associated mild and severe illness (DS2 in Table 1 and Appendix S1.) across the syndromes evaluated in this study.

In addition, we postulated that the hospitalization and outpatient consultation costs may be different (probably higher) among patients with influenza‐associated circulatory and non‐respiratory/non‐circulatory illness compared to those with influenza‐associated SARI or ILI. Hence, we implemented a sensitivity analysis by replacing the estimated cost of hospitalization and outpatient consultation among influenza‐positive patients with SARI or ILI by the cost per patient day equivalent (PDE; any hospitalization cost) and any outpatient consultation cost to obtain an estimate that would account for differential treatment costs across different clinical presentations (DS5 in Table 1 and Appendix S1).

#### 2.3.2 Indirect cost estimation

We estimated costs associated with absenteeism due to mild and severe episodes of influenza‐associated illness. For absenteeism due to hospitalization, number of days absent from work or school was estimated as the length of hospitalization plus 1.5 days as reported by Molinari et al.^34^ Absenteeism was estimated for affected patients and their caregivers, parametrized within syndromes using length of hospitalization obtained from DS3 and DS4 for patients (Table 1 and Appendix S1) and DS6 for caregivers (Table 1 and Appendix S1). For mild illness, number of productive days lost was obtained from DS6 and DS7 (Table 1 and Appendix S1).

School absenteeism due to mild or severe illness was calculated for individuals aged 5‐19 years, and work absenteeism was calculated for individuals aged 20‐64 years including patients and their caregivers. School and work absenteeism for hospitalized patients were adjusted based on five working/school days per week with work absenteeism for patients and caregivers further adjusted to account for the unemployment rate in South Africa (DS9 in Table 1 and Appendix S1). The mean absenteeism per illness episodes was obtained by dividing the days of absenteeism as estimated above (ie, excluding individuals of non‐school or working age, and adjusting for five working/school days per week and the unemployment rate) by the total number of illness episodes, including individuals of non‐school/working age. This resulted in the mean absenteeism for school or work per illness episode to be lower than the mean number of hospitalization days per illness episode.

We estimated the indirect cost of medically and non‐medically attended illness by multiplying minimum daily wages by the number of days absent from work as estimated above for patient and caregiver. We only considered value of lost time from paid activities but did not consider value of time for unpaid activities such as housekeeping or leisure.

### 2.4 Estimation of years of life lost due to influenza‐associated deaths

We estimated the YLL due to influenza‐associated deaths using the national estimates of influenza‐associated deaths (DS2 in Table 1 and Appendix S1) and the life expectancy for South Africa (DS10 in Table 1 and Appendix S1). YLL was estimated for all influenza‐associated deaths as well as for influenza‐associated SARI deaths.

### 2.5 Ethical approval

The SARI protocol (DS2) was approved by the University of the Witwatersrand Human Research Ethics Committee (HREC) and the University of KwaZulu‐Natal Human Biomedical Research Ethics Committee (BREC) protocol numbers M081042 and BF157/08, respectively. The ILI protocol (DS2) was approved by HREC and BREC protocol numbers M120133 and BF080/12, respectively. This surveillance was deemed non‐research by the US Centers for Disease Control and Prevention (non‐research determination number: 2012‐6197). The costing protocol (DS6) was approved by the HREC protocol number M121195. All other data sources were publically available.

## 3 RESULTS

### 3.1 Influenza virus surveillance among patients with ILI or SARI and length of hospitalization among patients with respiratory, circulatory, and non‐respiratory/non‐circulatory illness (DS3 and DS4)

In 2013‐2015, we enrolled and tested 13 912 patients at selected sentinel sites, of which 5525 (39.7%) presented with ILI and 8387 (60.3%) presented with SARI. Among these, influenza viruses were detected in 7.9% (1106/13 912) of specimens; 12.4% (683/5525) in patients presenting with ILI; and 5.0% (423/8387) in patients presenting with SARI. The mean length of hospitalization was 5.5 days among any patient with SARI and 5.3 days among laboratory influenza‐positive patients with SARI (*P* = 0.356) (DS3).

During the same period, the mean length of hospitalization was 5.7, 7.8, and 4.9 days among patients hospitalized at a large, private hospital network (NetCare) with respiratory, circulatory, or non‐respiratory/non‐circulatory illness, respectively (DS4).

### 3.2 Direct medical and non‐medical costs incurred by the patient/caregiver and absenteeism due to medically attended illness (DS5 and DS6)

In 2014, we enrolled 1788 patients into a costing study at selected influenza surveillance sentinel sites, of which 527 (29.5%) presented with ILI and 1261 (70.5%) presented with SARI. Of these, 15.2% (80/527) and 5.4% (68/1261) tested positive for influenza viruses, respectively. The mean monthly household income, including government support grants, was $559 (median: $475; 2.5th‐97.5th percentiles: $191‐$1156). Average direct out‐of‐pocket costs and number of days absent from school or work are provided in Table 2, while the itemized unit costs for patients with influenza‐associated SARI or ILI are provided in Table 3.

**Table 2:** Direct out‐of‐pocket costs and days of absenteeism for medically attended mild or severe respiratory illness, South Africa, 2014 (DS6)

Category	Mean value per illness episode (95% CI)			
	Any ILI	Influenza‐associated ILI	Any SARI	Influenza‐associated SARI
Proportion of illness episodes attended by a caregiver (%)	52.0 (47.1‐56.9)	64.9 (52.9‐75.6)	96.2 (95.0‐97.3)	98.4 (91.6‐99.9)
Patient absenteeism (d)	0.7 (0.5‐0.9)	0.8 (0.2‐1.4)	2.1 (1.9‐2.3)	1.8 (1.2‐2.4)
Caregiver absenteeism (d)	0.5 (0.3‐0.7)	0.6 (0.2‐0.8)	1.4 (1.2‐1.6)	1.3 (0.7‐1.9)
Out‐of‐pocket cost for patient ($)	2.9 (1.0‐4.8)	1.5 (1.1‐1.9)	9.0 (7.9‐10.2)	6.6 (3.5‐9.6)
Medical	0.4 (0.1‐0.7)	0.2 (0.1‐0.3)	3.5 (2.7‐4.4)	3.3 (1.0‐5.6)
Non‐medical^a^	2.5 (0.8‐4.1)	1.3 (0.9‐1.6)	5.5 (4.7‐6.3)	3.3 (1.4‐5.1)
Out‐of‐pocket cost for caregiver ($)	1.7 (0.4‐3.1)	1.3 (0.8‐1.7)	12.1 (8.6‐15.5)	7.5 (5.1‐9.9)

Abbreviations: CI, confidence intervals; ILI, influenza‐like illness (outpatients); SARI, severe acute respiratory illness (inpatients).

a Non‐medical costs include cost for transportation and additional food.

**Table 3:** Healthcare facility costs for medically attended mild or severe respiratory illness, South Africa, 2013‐2015 (DS3 and DS5)

Item	Unit cost ($)	Any SARI			Influenza‐associated SARI		
		% of item^a^	Mean duration of incurred cost (d)	Mean cost per illness episode ($)	% of item	Mean duration of incurred cost (d)	Mean cost per illness episode ($)
Facility fee (24H)	84.2	100.0	5.5	463.1	100.0	5.3	446.2
Consultation (24H)	24.3	100.0	5.5	133.6	100.0	5.3	128.7
ICU (24H)	691.6	1.3	2.3	20.7	1.0	2.5	17.3
Chest X‐ray	26.2	78.3	N/A	20.4	79.1	N/A	20.7
Oxygen (24H)	34.3	38.1	1.3	16.9	35.2	1.2	14.4
Antibiotic treatment	18.3^b^	94.1	N/A	17.2	96.3	N/A	17.6
Other medications	8.0^c^	100.0	N/A	8.0	100.0	N/A	8.0
HIV test	17.6^d^	83.2	N/A	14.6	77.8	N/A	13.7
Bacterial culture	4.1	30.7	N/A	1.3	25.8	N/A	1.1
Tuberculosis test	25.0	37.1	N/A	9.3	37.1	N/A	9.3
Blood cell count	4.6	88.1	N/A	4.1	88.3	N/A	4.1
CRP test	5.9	47.4	N/A	2.8	43.1	N/A	2.5
ESR test	2.8	12.2	N/A	0.3	11.9	N/A	0.3
Urea test	2.4	74.2	N/A	1.8	73.2	N/A	1.8
Total	N/A	N/A	N/A	714.8	N/A	N/A	685.6
PDE	N/A	N/A	N/A	129.8	N/A	N/A	129.4

Item	Unit cost ($)	Any ILI			Influenza‐associated ILI		
		% of item	Mean duration of incurred cost (d)	Mean cost per illness episode ($)	% of item	Mean duration of incurred cost (d)	Mean cost per illness episode ($)
Facility fee	8.4	100.0	N/A	8.4	100.0	N/A	8.4
Consultation	8.1	100.0	N/A	8.9	100.0	N/A	8.9
Antibiotic treatment	11.3 a	40.1	N/A	4.5	0.37	N/A	4.2
Other medications	3.3 b	98.0	N/A	3.2	0.99	N/A	3.3
Total	N/A	N/A	N/A	25.1	N/A	N/A	24.8

Abbreviations: CRP, C‐reactive protein; ESR, erythrocyte sedimentation rate; HIV, human immunodeficiency virus; ICU, intensive care unit; ILI, influenza‐like illness; PDE, cost per patient day equivalent; SARI, severe acute respiratory illness.

a % of individuals that received the item.

b Calculated as the mean cost among patients that were prescribed antibiotics. It accounts for differential costs related to prescribed antibiotics and posology.

c Calculated as the mean cost among patients that were prescribed medications other than antibiotics. It accounts for differential costs related to prescribed medications and posology.

d Calculated as the mean cost among patients that were tested for HIV. It accounts for differential costs related to HIV diagnostics utilized (ie, rapid test, ELISA, or PCR) as well as for CD4+ cell count and viral load among HIV‐positive individuals when requested by the attending physician.

### 3.3 Economic burden of influenza‐associated illness

**Figure 1:**
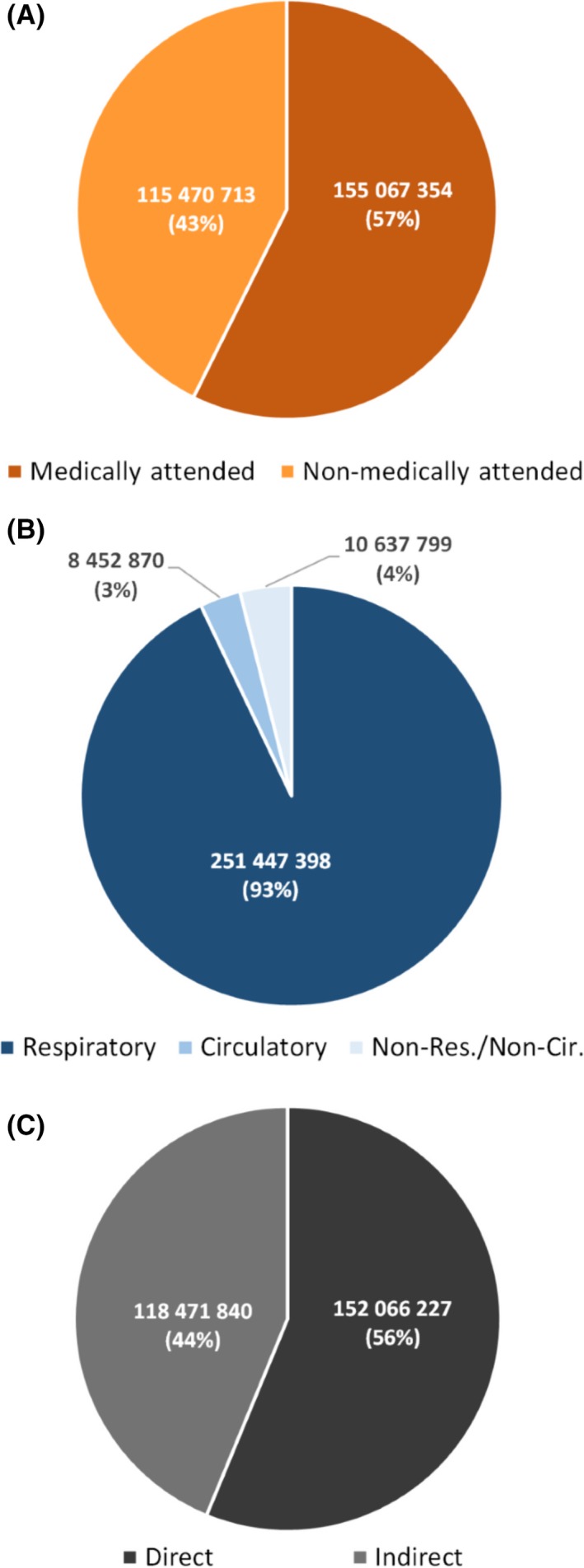
Estimated mean annual proportional cost (in $) of influenza‐associated illness in South Africa during 2013‐2015 by: A, healthcare attendance; B, syndrome; and C, cost type

The mean direct and indirect cost per illness episode was $25 overall; $770 for medically attended severe illness; $40 for medically attended mild illness; and $15 for non‐medically attended mild or severe illness. Mean annual cost of influenza‐associated illness was estimated at $270.5 million (Table 4), 0.08% ($5.1 per capita) of the total mean annual GDP. Of this, the direct cost for medically and non‐medically attended illness was $152.1 million (56% of the total cost) and the direct and indirect cost of medically attended influenza‐associated illness was $155.1 million (57% of the total cost) (Table 4 and Figure 1A,C). Of the direct cost, 73% ($111.3 million) was incurred by the healthcare system for medical care of medically attended influenza‐associated mild and severe illness and the remaining $40.7 million were out‐of‐pocket expenses for medically and non‐medically attended mild or severe illness. The indirect cost of influenza‐associated illness was $118.5 million (44% of the total cost). Of this indirect cost, 69% ($82.0 million) was from non‐medically attended illness.

**Table 4:** Mean annual estimates of the direct and indirect costs (expressed in US dollars) of influenza‐associated illness in South Africa, 2013‐2015 (main analysis using influenza‐associated illness specific costs)

Parameter	Mean value per illness episode (95% CI)	Total (95% CI)
Medically attended severe illness (hospitalization)		
Total absenteeism (d)	3.4 (2.3‐4.6)	209 572 (84 619‐440 568)
Absenteeism from school	0.5 (0.3‐0.7)	30 731 (11 922‐56 851)
Absenteeism from work^a^	2.9 (2.7‐3.1)	178 841 (103 728‐253 954)
Total cost ($)	770 (453‐1212)	47 079 788 (16 721 728‐115 933 018)
Direct cost	733 (433‐1154)	44 850 281 (15 994 271‐110 428 451)
Healthcare system	719 (425‐1135)	43 994 581 (15 689 490‐108 586 025)
Out‐of‐pocket^a^	14 (8‐19)	855 700 (304 781‐1 842 426)
Indirect cost^a^	36 (20‐58)	2 229 507 (727 457‐5 504 567)
Medically attended mild illness (outpatient consultation)		
Total absenteeism (d)	1.4 (0.9‐2.1)	3 742 312 (873 845‐6 817 553)
Absenteeism from school	1.0 (0.8‐1.2)	2 521 551 (920 848‐3 920 389)
Absenteeism from work^a^	0.4 (0.3‐0.5)	1 220 761 (878 948‐1 562 574)
Total cost ($)	40 (34‐53)	107 987 566 (35 258 178‐174 171 071)
Direct cost	27 (24‐30)	73 787 330 (29 030 921‐97 863 181)
Healthcare system	25 (23‐27)	67 298 918 (27 162 744‐87 393 369)
Out‐of‐pocket^a^	2 (1‐3)	6 488 412 (1 868 177‐10 469 812)
Indirect cost^a^	13 (6‐23)	34 200 236 (6 227 257‐76 307 890)
Non‐medically attended mild and severe illness		
Total absenteeism (d)	1.1 (0.9‐1.7)	9 272 992 (2 615 225‐16 256 362)
Absenteeism from school	0.5 (0.6‐0.7)	4 243 098 (1 730 659‐6 488 287)
Absenteeism from work^a^	0.6 (0.3‐1.0)	5 029 894 (884 566‐9 768 075)
Total cost ($)	15 (9‐24)	115 470 713 (28 367 196‐227 359 471)
Direct cost	4 (3‐5)	33 428 616 (13 598 817‐44 927 770)
Healthcare system	Not applicable	Not applicable
Out‐of‐pocket^a^	4 (3‐5)	33 428 616 (13 598 817‐44 927 770)
Indirect cost^a^	11 (5‐19)	82 042 097 (14 768 379‐182 431 701)
Total of above categories		
Total absenteeism (d)	1.2 (0.9‐1.8)	13 224 876 (3 573 689‐23 514 483)
Absenteeism from school	0.6 (0.4‐0.8)	6 795 380 (2 663 429‐10 465 527)
Absenteeism from work^a^	0.6 (0.3‐0.9)	6 429 496 (1 867 242‐11 584 603)
Total cost ($)	25 (20‐32)	270 538 067 (80 347 102‐517 463 560)
Direct cost	14 (10‐19)	152 066 227 (58 624 009‐253 219 402)
Healthcare system	10 (6‐15)	111 293 499 (42 852 234‐195 979 394)
Out‐of‐pocket^a^	4 (3‐5)	40 772 728 (15 771 775‐57 240 008)
Indirect cost^a^	11 (5‐17)	118 471 840 (21 723 093‐264 244 158)

Abbreviation: CI, confidence intervals.

a Patient and caregiver.

Cost due to influenza‐associated respiratory, circulatory, and non‐respiratory/non‐circulatory illness accounted for 93% ($251.4 million), 3% ($8.4 million), and 4% ($10.6 million) of the total cost, respectively (Table 5 and Figure 1B). The cost of influenza‐associated WHO‐defined SARI and ILI (a subset of all‐respiratory illness) was $95.3 million accounting for 35% of the total cost (Table S3). This cost using the unmodified WHO toolkit (ie, not accounting for direct and indirect cost of non‐medically attended influenza‐associated SARI and the indirect cost of non‐medically attended influenza‐associated ILI) was estimated at $87.6 million (Table S3).

**Table 5:** Mean annual estimates of the direct and indirect cost of influenza‐associated illness by syndrome in South Africa, 2013‐2015 (main analysis using influenza‐associated illness specific costs)

Parameter	Respiratory value (95% CI)	Circulatory value (95% CI)	Non‐respiratory/non‐circulatory value (95% CI)
Medically attended severe illness (hospitalization)			
Total absenteeism (d)	132 259 (57 588‐250 458)	34 828 (10 664‐88 764)	42 485 (16 367‐101 346)
Absenteeism from school	23 523 (8022‐42 318)	1413 (405‐3477)	5795 (3495‐11 056)
Absenteeism from work^a^	108 736 (63 067‐154 405)	33 415 (19 381‐47 449)	36 690 (21 280‐52 100)
Total cost ($)	29 615 038 (11 024 277‐64 591 790)	7 951 535 (2 564 349‐25 204 526)	9 513 216 (3 133 104‐26 136 702)
Direct cost	28 208 096 (10 529 730‐61 462 487)	7 580 920 (2 471 988‐24 095 517)	9 061 265 (2 992 553‐24 870 447)
Healthcare system	27 638 889 (10 316 551‐60 350 017)	7 477 273 (2 440 971‐23 815 716)	8 878 419 (2 931 968‐24 420 292)
Out‐of‐pocket^a^	569 207 (213 179‐1 112 470)	103 647 (31 017‐279 801)	182 846 (60 585‐450 155)
Indirect cost^a^	1 406 942 (494 547‐3 129 303)	370 614 (92 360‐1 109 009)	451 951 (140 550‐1 266 255)
Medically attended mild illness (outpatient consultation)			
Total absenteeism (d)	3 730 033 (870 285‐6 789 121)	2478 (949‐6419)	9801 (2611‐22 013)
Absenteeism from school	2 519 832 (920 215‐3 916 914)	439 (218‐943)	1280 (415‐2532)
Absenteeism from work^a^	1 210 201 (871 345‐1 549 057)	2039 (1468‐2610)	8521 (6135‐10 907)
Total cost ($)	107 633 247 (35 114 554‐173 444 720)	71 506 (38 272‐163 983)	282 814 (105 352‐562 368)
Direct cost	73 545 226 (28 912 663‐97 455 060)	48 859 (31 513‐92 138)	193 245 (86 745‐315 983)
Healthcare system	67 078 103 (27 052 096‐87 028 910)	44 563 (29 485‐82 281)	176 252 (81 163‐282 178)
Out‐of‐pocket^a^	6 467 123 (1 860 567‐10 426 150)	4296 (2028‐9857)	16 993 (5582‐33 805)
Indirect cost^a^	34 088 021 (6 201 890‐75 989 661)	22 646 (6760‐71 844)	89 569 (18 607‐246 385)
Non‐medically attended mild and severe illness			
Total absenteeism (d)	9 170 313 (2 579 963‐15 989 327)	37 653 (11 762‐99 659)	65 026 (23 500‐167 376)
Absenteeism from school	4 232 092 (1 724 301‐6 463 954)	1808 (822‐5107)	9198 (5536‐19 226)
Absenteeism from work^a^	4 938 221 (855 662‐9 525 373)	35 845 (10 940‐94 552)	55 828 (17 964‐148 150)
Total cost ($)	114 199 113 (27 963 705‐223 455 309)	429 831 (126 957‐1 344 509)	841 769 (276 534‐2 559 655)
Direct cost	33 205 995 (13 496 244‐44 319 798)	58 625 (28 199‐173 833)	163 996 (74 374‐434 139)
Healthcare system	Not applicable	Not applicable	Not applicable
Out‐of‐pocket^a^	33 205 995 (13 496 244‐44 319 798)	58 625 (28 199‐173 833)	163 996 (74 374‐434 139)
Indirect cost^a^	80 993 118 (14 467 461‐179 135 510)	371 206 (98 758‐1 170 675)	677 773 (202 160‐2 125 516)
Total of above categories			
Total absenteeism (d)	13 032 605 (3 507 836‐23 028 906)	74 959 (23 375‐194 842)	117 312 (42 478‐290 735)
Absenteeism from school	6 775 447 (2 652 538‐10 423 186)	3660 (1445‐9527)	16 273 (9446‐32 814)
Absenteeism from work^a^	6 257 158 (1 790 074‐11 228 835)	71 299 (31 789‐144 611)	101 039 (45 379‐211 157)
Total cost ($)	251 447 398 (74 102 535‐461 491 819)	8 452 870 (2 729 578‐26 713 016)	10 637 799 (3 514 989‐29 258 725)
Direct cost	134959317 (52 938 637‐203 237 345)	7 688 404 (2 531 700‐24 361 488)	9 418 506 (3 153 672‐25 620 569)
Healthcare system	94 716 992 (37 368 647‐147 378 927)	7 521 836 (2 470 456‐23 897 997)	9 054 671 (3 013 131‐24 702 470)
Out‐of‐pocket^a^	40 242 325 (15 569 990‐55 858 418)	166 568 (61 244‐463 491)	363 835 (140 541‐918 099)
Indirect cost^a^	116 488 081 (21 163 898‐258 254 474)	764 466 (197 878‐2 351 528)	1 219 293 (361 317‐3 638 156)

Abbreviations: CI: confidence intervals

For this category, severe and mild cases are those meeting the World Health Organization (WHO) severe acute respiratory illness (SARI) and influenza‐like illness (ILI) case definitions, respectively. This is a subset of all‐respiratory illness.

a Patient and caregiver.

The cost of influenza‐associated illness using the PDE was estimated at $292.4 million (Table S1). The cost by syndromes using the PDE is provided in Tables S2 and S4.

### 3.4 Absenteeism for influenza‐associated illness

**Figure 2:**
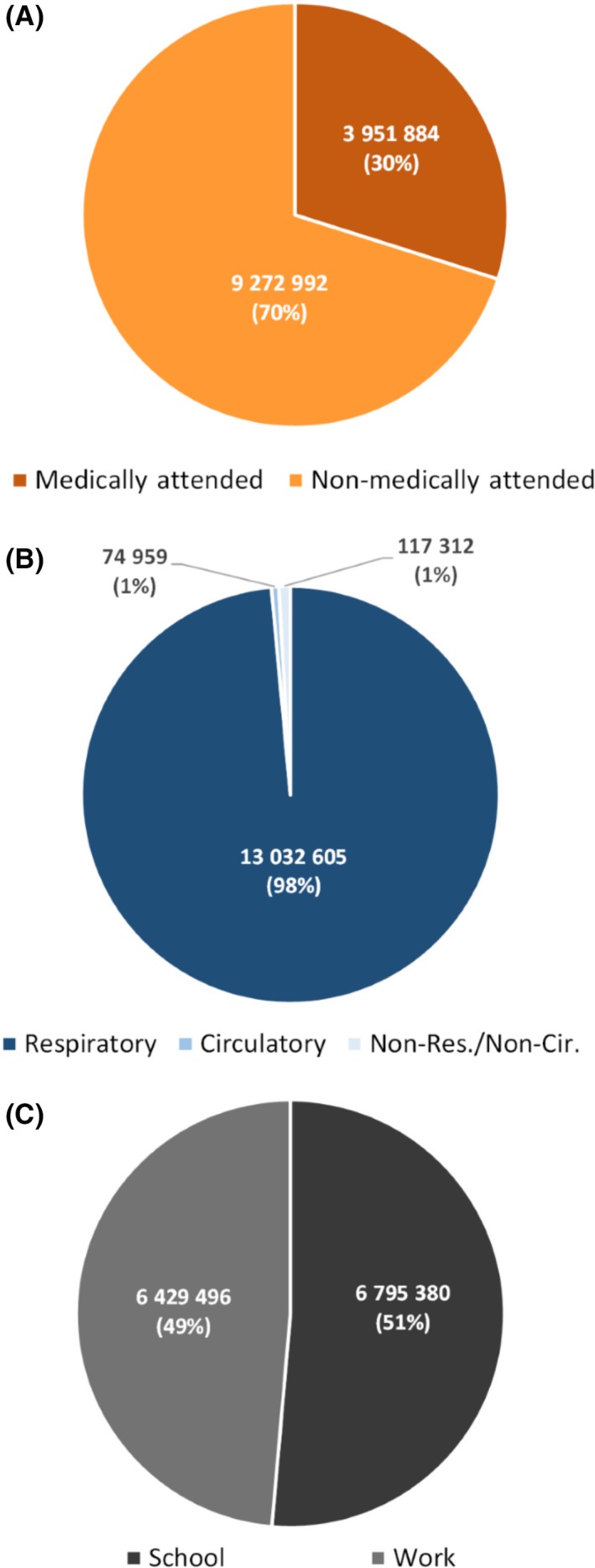
Estimated mean annual proportional number of absenteeism (in days) from influenza‐associated illness in South Africa during 2013‐2015 by: A, healthcare attendance; B, syndrome; and C, absenteeism type

The mean annual absenteeism associated with influenza‐associated illness was estimated at 13.2 million days (1.2 days per illness episode; Table 4); 6.7 million days of absenteeism from school (51%; 0.6 days per illness episode); and 6.4 million days of absenteeism from work (49%; 0.6 days per illness episode; Table 4 and Figure 2C). Absenteeism due to non‐medically attended influenza‐associated illness accounted for 70% (9.2 million days) of total absenteeism (Table 4 and Figure 2A).

Absenteeism due to influenza‐associated respiratory, circulatory, and non‐respiratory/non‐circulatory illness accounted for 98% (13.0 million days), 1% (74 959 days), and 1% (117 312 days) of the total absenteeism, respectively (Table 5 and Figure 2B). Absenteeism for influenza‐associated SARI and ILI (a subset of all‐respiratory illness) was 4.5 million days accounting for 34% of the total absenteeism (Table S3). The absenteeism for influenza‐associated illness using the unmodified WHO toolkit was 3.2 million days (Table S3).

### 3.5 Years of life lost for influenza‐associated death

During 2013‐2015, the estimated mean annual YLL for influenza‐associated death was estimated at 304 867 (Table 6). The YLL due to influenza‐associated respiratory, circulatory, and non‐respiratory/non‐circulatory deaths accounted for 34% (103 265 years), 16% (48 976 years), and 50% (152 626 years) of the total YLL, respectively (Table 6). The YLL for influenza‐associated SARI deaths (a subset of all‐respiratory deaths) was 65 697; 21% of the total YLL (Table S5).

**Table 6:** Estimated mean annual years of life lost (YLL) from influenza‐associated death by syndrome in South Africa, 2013‐2015

Age (in y)	Respiratory YLL (95% CI)	Circulatory YLL (95% CI)	Non‐respiratory/non‐circulatory YLL (95% CI)	Total YLL (95% CI)
<1	22 019 (15 091‐30 183)	1484 (1175‐3525)	35 378 (18 926‐46 573)	58 881 (35 192‐80 281)
1‐4	8814 (5855‐12 466)	2078 (1826‐2518)	6611 (2392‐7618)	17 503 (10 073‐22 602)
5‐19	6256 (2909‐10 702)	1427 (823‐3238)	14 653 (12 787‐25 245)	22 336 (16 519‐39 185)
20‐44	32 397 (22 600‐45 311)	9908 (5492‐15 660)	72 884 (47 389‐135 526)	115 189 (75 481‐196 497)
45‐64	22 197 (14 049‐32 172)	17 241 (10 983‐44 037)	15 918 (7686‐30 429)	55 356 (32 718‐106 638)
≥65	11 582 (5239‐19 568)	16 838 (6548‐29 541)	7182 (2842‐15 074)	35 602 (14 629‐64 183)
<5	30 833 (20 946‐42 649)	3562 (3001‐6043)	41 989 (21 318‐54 191)	76 384 (45 265‐102 883)
≥5	72 432 (44 797‐107 753)	45 414 (23 846‐92 476)	110 637 (70 704‐206 274)	228 483 (139 347‐406 503)
All	103 265 (65 743‐150 402)	48 976 (26 847‐98 519)	152 626 (9022‐260 465)	304 867 (184 612‐509 386)

## 4 DISCUSSION

We estimated the economic burden, absenteeism, and YLL from influenza‐associated illness in a middle‐income country in Africa. The estimated mean annual economic burden was substantial ($270.5 million) with direct and indirect costs contributing almost equally to the total cost and the cost of non‐medically attended illness making up 43% of the total economic burden. The economic burden incurred by the healthcare system to treat medically attended illness accounted for 41% ($111.3 million) of the total cost. The estimated mean annual length of absenteeism from school or work due to influenza‐associated illness was also substantial (13.2 million days) with absenteeism from non‐medically attended illness making up 70% of total absenteeism. Estimates obtained among individuals meeting the WHO SARI or ILI case definitions (as suggested in the WHO manual for estimating the economic burden of seasonal influenza^32^) underestimated the total economic burden by 65%, absenteeism by 66%, and YLL by 79%.

Whereas there are no studies on the economic burden of influenza‐associated illness from other African countries, the proportional cost of influenza‐associated illness to the GDP in South Africa (0.08%) was similar to those estimated from studies conducted in high‐ or middle‐income countries in North America, Europe, Asia, and Australia (range: 0.01%‐0.14%).^35^ The cost per capita in South Africa ($5.1) was lower than estimates in the United States and European countries (range: $27‐$63), but it was similar to those from middle‐income Asian countries (range: $1‐3).^35^

In our setting, the indirect cost of influenza‐associated illness accounted for 44% of the total cost. This was similar to studies conducted in the United States and Asia (range: 13%‐56%), whereas in Europe, the indirect cost accounted for a higher proportion of the total cost (range: 88%‐92%).^35^ Some of these differences may be related to different unit costs in diverse settings as well as dissimilar estimation approaches.

Whereas the cost per illness episode of non‐medically attended illness ($15) was lower than that for medically attended illness, the cost of non‐medically attended illness accounted for 43% of the total cost. This is because a large proportion of influenza‐associated mild (~74%) and severe (~56%) illness is not medically attended in South Africa.^6^ This was also reflected in the high proportional contribution of absenteeism from non‐medically attended illness (70%) to the total.

Irrespective of syndromes, the cost of influenza‐associated medically attended mild illness accounted for 70% of the cost of any medically attended illness (severe and mild). Whereas the cost of and absenteeism from influenza‐associated severe illness per episode are higher than those of mild illness, the number of severe illness episodes is substantially lower (1.2% of total illness) than the number of mild illness episodes.^6^ The cost of and absenteeism from influenza‐associated respiratory illness accounted for 93% and 98% of the total, respectively. Influenza‐associated non‐respiratory illness accounts for a large proportion of influenza‐associated severe illness (45%), but a small proportion of influenza‐associated mild illness (<1%)^6^ resulting in an overall minimal contribution of influenza‐associated non‐respiratory illness to the total cost (7%) and absenteeism (2%).

We found a substantial underestimation of the total cost, absenteeism, and YLL when restricting the analysis to individuals with influenza‐associated SARI and ILI, a subset of all‐respiratory severe and mild illness, respectively. This is because, in our setting, influenza‐associated ILI, influenza‐associated non‐fatal SARI, and influenza‐associated SARI deaths represent only 34%, 40%, and 20% of the total number of influenza‐associated mild, severe‐non‐fatal, and fatal illness, respectively.^6^ In addition, when we implemented the analysis among individuals with influenza‐associated SARI and ILI using the unmodified WHO tool (ie, not accounting for direct and indirect costs of non‐medically attended influenza‐associated SARI and the indirect cost of non‐medically attended influenza‐associated ILI), we further underestimated cost by $7.7 million and absenteeism by 1.3 million days.

Our study has some limitations that warrant discussion. First, we did not include the indirect cost associated with deaths including lost productivity because such estimates are not available for South Africa. Second, because we did not have specific estimates of costs for influenza‐associated all‐circulatory and non‐respiratory/non‐circulatory illness, we assumed the same costs as for influenza‐associated respiratory illness. However, given the proportionally small number of influenza‐associated non‐respiratory illness (<1% of the total illness episodes), this category minimally influenced the total cost estimates. In addition, the economic burden estimates using the PDE costs (sensitivity analysis; $292.4 million) and the influenza‐associated illness specific cost (main analysis; $270.5 million) were similar. Last, because of the high disparity of wages in South Africa, we used the minimum wages to estimate indirect cost; hence, this represents a minimum estimate.

In conclusion, we found a substantial economic burden, absenteeism, and YLL associated with influenza disease in South Africa, with influenza‐associated mild and non‐medically attended illness accounting for a large proportion of total cost and absenteeism in our setting. The economic burden was high from both a government and a societal perspective. Estimates obtained from individuals meeting the SARI and ILI case definition as suggested in the WHO manual for estimating the economic burden of seasonal influenza^32^ would underestimate the total burden in excess of 60% for each of the three endpoints evaluated in this study. This study provides the foundation for future cost‐benefit analysis on potential interventions such as influenza immunization. However, despite the substantial disease^6^ and economic burden of influenza‐associated illness in South Africa, given the competing health priorities in the country, it is unlikely that the South African government will introduce universal influenza immunization in the near future. Cost‐burden and cost‐benefit studies on the potential impact of influenza immunization among groups at increased risk of influenza‐associated severe illness, including different age groups, are warranted to further guide the current influenza vaccination guidelines^12^ and refine the existing influenza policy.^13^ An alternative strategy to risk group‐based vaccination is to target the community transmitters of infection, such as school‐aged children, thereby potentially reducing overall community disease burden and associated cost.^36^, ^37^ Cost‐benefit studies of these approaches are also warranted.

## ETHICS

The SARI protocol (DS2 in Table 1) was approved by the University of the Witwatersrand Human Research Ethics Committee (HREC) and the University of KwaZulu‐Natal Human Biomedical Research Ethics Committee (BREC) protocol numbers M081042 and BF157/08, respectively. The ILI protocol (DS2 in Table 1) was approved by BREC protocol number BF080/12. This surveillance was deemed non‐research by the US Centers for Disease Control and Prevention (non‐research determination number: 2012‐6197). The costing protocol (DS6 in Table 1) was approved by the HREC protocol number M121195. All other data sources were publically available.

## CONFLICT OF INTEREST

All authors declare that they have no commercial or other associations that may pose a conflict of interest.

## AUTHOR CONTRIBUTIONS

All authors had full access to the data in the study and take responsibility for the integrity of the data and the accuracy of the data analysis. Tempia S, Moyes J, Cohen AL, and Cohen C conceived and designed the study. Tempia S, Moyes J, Cohen AL, Walaza S, Teurnicht FK, Hellferscee O, Wolter N, von Gottberg A, Nguweneza A, McAnerney JM, Dawood H, Variava E, and Cohen C acquired, analyzed, or interpreted the data. Tempia S drafted the manuscript. Tempia S, Moyes J, Cohen AL, Walaza S, Edoka I, McMorrow ML, Treurnicht FK, Hellferscee O, Wolter N, von Gottberg A, Nguweneza A, McAnerney JM, Dawood H, Variava E, and Cohen C critically revised the manuscript for important intellectual content.

## DISCLAIMER

The findings and conclusions in this report are those of the authors and do not necessarily represent the official position of the US Centers for Disease Control and Prevention, USA, or the National Institute for Communicable Diseases, South Africa.
