# Supplementary material for: Health and economic burden of influenza‐associated illness in South Africa, 2013‐2015
Source: Influenza Other Respir Viruses. 2019 Jun 11;13(5):484–95. doi: 10.1111/irv.12650 (PMC6692552; doi:10.1111/irv.12650)
Supplement: Supplementary file 1 [file IRV-13-484-s001.docx]

**Health and Economic Burden of Influenza-Associated Illness in South Africa, 2013-2015 (Supplementary Material)**

**Authors**

Stefano Tempia^1,2,3,4^, Jocelyn Moyes^3,5^, Adam L. Cohen^1,2,6^, Sibongile Walaza^3,5^, Ijeoma Edoka^7^, Meredith L. McMorrow^1,2^, Florette K. Treurnicht^3^, Orienka Hellferscee^3,8^, Nicole Wolter^3,8^, Anne von Gottberg^3,8^, Athermon Nguweneza^3^, Johanna M. McAnerney^3^, Halima Dawood^9,10^, Ebrahim Variava^11,12,13^, Cheryl Cohen^3,5^

**Affiliations**

^1^Influenza Division, Centers for Disease Control and Prevention, Atlanta, Georgia, United States of America.

^2^Influenza Program, Centers for Disease Control and Prevention, Pretoria, South Africa.

^3^Centre for Respiratory Diseases and Meningitis, National Institute for Communicable Diseases of the National Health Laboratory Service, Johannesburg, South Africa.

^4^MassGenics, Duluth, Georgia, Unites States of America.

^5^School of Public Health, Faculty of Health Sciences, University of the Witwatersrand, Johannesburg, South Africa.

^6^Expanded Programme on Immunization, Department of Immunization, Vaccines and Biological, World Health Organization, Geneva, Switzerland.

^7^ Priority Cost Effectiveness Lessons for System Strengthening South Africa, South Africa Medical Research Council, Wits Center for Health Economic and Decision Science, School of Public Health, University of the Witwatersrand, Johannesburg, South Africa.

^8^School of Pathology, Faculty of Health Sciences, University of the Witwatersrand, Johannesburg, South Africa.

^9^Department of Medicine, Greys Hospital, Pietermaritzburg, South Africa.

^10^Caprisa, University of KwaZulu-Natal, Pietermaritzburg, South Africa.

^11^Department of Medicine, Klerksdorp-Tshepong Hospital Complex, Klerksdorp, South Africa.

^12^Department of Medicine, Faculty of Health Sciences, University of the Witwatersrand, Johannesburg, South Africa.

^13^Perinatal HIV Research Unit, University of the Witwatersrand, Johannesburg, South Africa.

**METHODS**

**Data sources**

*Data source 1 (DS1): Population denominators*

We obtained age- and year-specific population denominators from projections of 2011 census data for South Africa [^[[1]](#endnote-1)^,^[[2]](#endnote-2)^,^[[3]](#endnote-3)^]. South Africa had an estimated population of 54,860,530 individuals in 2015 of which 5,827,421 (10.7%) were children aged <5 years.

*Data source 2 (DS2): National estimates of numbers and rates of medically- and non-medically-attended influenza-associated mild and severe illness and deaths.*

We obtained national estimates of influenza-associated illness for South Africa during 2013-2015 from a previously published study [^[[4]](#endnote-4)^]. This includes estimates of medically- and non-medically-attended influenza-associated mild and severe illness and death among patients with respiratory, circulatory and non-respiratory/non-circulatory clinical presentation. Estimates among patients meeting the WHO influenza-like illness (ILI) and severe acute respiratory illness (SARI) case definitions (a subset of all-respiratory mild and severe illness and deaths) were also available. The estimated mean annual number of influenza-associated illness episodes was 10,737,847 (rate: 19,849.4 per 100,000 population). Of these episodes, 10,598,138 (98.7%; rate: 19,591.1 per 100,000 population), 128,173 (1.2%; rate: 236.9 per 100,000 population) and 11,536 (0.1%; rate: 21.3 per 100,000 population) were mild, severe-non-fatal and fatal, respectively.

*Data source 3 (DS3): Laboratory-confirmed influenza surveillance among inpatients with SARI and outpatients with ILI at selected surveillance sites during 2013-2015.*

We obtained data on the length of hospitalization and procedures during admission [e.g., admission to the intensive care unit (ICU), chest X-rays, oxygen support, medications and laboratory tests] for influenza-associated SARI from laboratory-confirmed influenza surveillance conducted at seven hospitals situated in four out of nine provinces (Gauteng, KwaZulu-Natal, North West and Mpumalanga) during 2013-2015. Influenza surveillance among outpatients with ILI was also implemented at two clinics situated in the same catchment area of the hospitals located in KwaZulu-Natal and North West Provinces. The procedures of these surveillance programs have been previously described [^[[5]](#endnote-5)^,^[[6]](#endnote-6)^]. Briefly, trained surveillance nurses completed case report forms that included demographic, clinical and epidemiological information for all enrolled cases. SARI patients were followed up until discharge, transfer or in-hospital death and procedures were recorded. In addition, respiratory specimens (i.e., nasopharyngeal aspirates for children aged <5 years and nasopharyngeal and oropharyngeal swabs from persons aged ≥5 years) were collected from all enrolled patients, placed in the same vial containing universal transport medium, stored at 4-8°C and transported to the National Institute for Communicable Diseases, Johannesburg, South Africa within 72 hours of collection for testing. Specimens were tested for influenza A and B viruses using a real-time reverse transcription polymerase chain reaction assay [^[[7]](#endnote-7)^].

*Data source 4 (DS4): Hospitalization data from large private hospital network (NetCare) active in 7/9 provinces during 2013-2015.*

We obtained data on the length of hospitalization for all-circulatory and non-respiratory/non-circulatory illness from a large private hospital network (NetCare) active in 7/9 provinces that collects data on all hospitalizations coded according to the *International Classification of Diseases, 10^th^* *revision* (ICD-10) [^[[8]](#endnote-8)^].

*Data source 5 (DS5): Cost of hospitalization and outpatient consultation*

We obtained itemized unit costs of hospitalization/procedures (e.g. facility, consultation, intensive care unit, chest X-ray and oxygen), medications and laboratory testing from the uniform patient fee schedule of the National Department of Health and the state price list of the National Health Laboratory Service [^[[9]](#endnote-9)^,^[[10]](#endnote-10)^,^[[11]](#endnote-11)^,^[[12]](#endnote-12)^]. In addition, we obtained national estimates of the cost of hospitalization (cost per patient day equivalent – PDE) and outpatient consultation during 2013-2015 from District Health Barometer Reports [^[[13]](#endnote-13)^,^[[14]](#endnote-14)^]. Costs per PDE were calculated by dividing the total expenditures for the hospital (including medical care, treatment, diagnostics, procedures, etc.) by the number of PDEs. PDEs were calculated by adding the number of inpatient days plus one-half day of inpatient plus one-third of an outpatient visit and one-third of an emergency room visit. Outpatient consultation costs were calculated by dividing the money spent on non-hospital primary health care (PHC) by the total PHC headcount. The estimated mean national costs per PDE and outpatient consultation during 2013-2015 were $183 (2,053 Rands) and $26 (292 Rands), respectively.

*Data source 6 (DS6): Costing study of laboratory-confirmed influenza inpatients with SARI and outpatients with ILI at selected surveillance sites during 2014.*

We implemented a costing survey among inpatients with SARI and outpatients with ILI in the same facilities where influenza surveillance was conducted (DS3) during January to December 2014. We aimed to co-enroll in the costing study 2 inpatients with SARI and 2 outpatients with ILI per week per facility within the following age categories: <1, 1-4, 5-19, 20-44, 45-64 and ≥65 years. Data collected included patient out-of-pocket hospitalization or outpatient consultation costs not included in the hospital of clinic fees, the direct non-medical costs (e.g., transportation and lodging for patient and caregiver) and absenteeism from school or work.

*Data source 7 (DS7): Community costing survey implemented in 4/9 provinces of South Africa*

We obtained estimates of direct medical costs (i.e., over-the-counter medications and consultation with traditional healers), direct non-medical costs (i.e., transportation), indirect costs (e.g., lost earning due to illness for patient and caregiver) and length of absenteeism from school or work for non-medically-attended respiratory illness from a community survey conducted in 2013 in 4/9 provinces of South Africa [^[[15]](#endnote-15)^].

*Data source 8 (DS8): Healthcare utilization survey among individuals with SARI or ILI in three South African communities.*

We obtained estimates on healthcare utilization among patients with SARI and ILI from healthcare utilization surveys conducted in three South African communities in 2012 and 2013 [^[[16]](#endnote-16)^,^[[17]](#endnote-17)^]. These data were used to assess the proportion of individuals with non-medically-attended illness that sought care from pharmacies or traditional healers.

*Data source 9 (DS9): Average wage and unemployment.*

We obtained the minimum daily wage for South Africa from reports from Treasury [^[[18]](#endnote-18)^] and the unemployment rate for individuals aged 20-64 years from Statistics South Africa [^[[19]](#endnote-19)^]. The minimum daily wage during the study period was $10.7 (120 Rands) and the unemployment rate was 26.4%.

*Data Source 10 (DS10): Life expectancy*

We obtained annual estimates of life expectancy for South Africa during the study period from WHO life tables [^[[20]](#endnote-20)^]. The mean annual life expectancy for children aged <1 year over the study period was 61.8 years.

**RESULTS**

**Table S1:** Estimated mean annual economic burden of influenza-associated illness in South Africa, 2013-2015 (sensitivity analysis using PDE and overall consultation costs).

| **Parameter** | **Mean value per illness episode (95% CI)** | **Total (95% CI)** |
| --- | --- | --- |
| **Medically-attended severe illness (hospitalization)** | | |
| **Total Cost ($)** | **1,070 (646-1,524)** | **65,422,757 (23,851,472-145,825,490)** |
| Direct Cost | 1,033 (626-1,467) | 63,193,250 (23,124,015-140,320,923) |
| Healthcare system | 1,019 (618-1,448) | 62,337,550 (22,819,234-138,478,497) |
| Out-of-pocket^a^ | 14 (8-19) | 855,700 (304,781-1,842,426) |
| Indirect cost^a^ | 36 (20-58) | 2,229,507 (727,457-5,504,567) |
| **Medically-attended mild illness (outpatient consultation)** | | |
| **Total Cost ($)** | **41 (35-55)** | **111,555,162 (35,960,375-182,432,135)** |
| Direct Cost | 28 (24-32) | 77,354,926 (29,733,118-106,124,245) |
| Healthcare system | 26 (23-29) | 70,866,514 (27,864,941-95,654,433) |
| Out-of-pocket^a^ | 2 (1-3) | 6,488,412 (1,868,177-10,469,812) |
| Indirect cost^a^ | 13 (6-23) | 34,200,236 (6,227,257-76,307,890) |
| **Non-medically-attended mild and severe illness** | | |
| **Total Cost ($)** | **15 (9-24)** | **115,470,713 (28,367,196-227,359,471)** |
| Direct Cost | 4 (3-5) | 33,428,616 (13,598,817-44,927,770) |
| Healthcare system | Not applicable | Not applicable |
| Out-of-pocket^a^ | 4 (3-5) | 33,428,616 (13,598,817-44,927,770) |
| Indirect cost^a^ | 11 (5-19) | 82,042,097 (14,768,379-182,431,701) |
| **Total** | | |
| **Total Cost ($)** | **27 (22-32)** | **292,448,632 (88,179,043-555,617,096)** |
| Direct Cost | 16 (10-22) | 173,976,792 (66,455,950-291,372,938) |
| Healthcare system | 12 (6-18) | 133,204,064 (50,684,175-234,132,930) |
| Out-of-pocket^a^ | 4 (3-5) | 40,772,728 (15,771,775-57,240,008) |
| Indirect cost^a^ | 11 (5-17) | 118,471,840 (21,723,093-264,244,158) |

Abbreviations: CI: confidence intervals; PDE: patient per day equivalent.

^a^ Patient and caregiver.

**Table S2:** Estimated mean annual economic burden of influenza-associated illness by syndrome in South Africa, 2013-2015 (sensitivity analysis using PDE and overall consultation costs).

| **Parameter** | **All-respiratory**  **Value (95% CI)** | **All-circulatory**  **Value (95% CI)** | **Non-respiratory/**  **non-circulatory**  **Value (95% CI)** |
| --- | --- | --- | --- |
| **Medically-attended severe illness (hospitalization)** | | | |
| **Total Cost ($)** | **41,138,713 (15,712,406-81,205,449)** | **11,069,087 (3,673,594-31,760,716)** | **13,214,957 (4,465,472-32,859,325)** |
| Direct Cost | 39,731,772 (15,217,859-78,076,146) | 10,698,472 (3,581,234-30,651,707) | 12,763,006 (4,324,922-31,593,070) |
| Healthcare system | 39,162,565 (15,004,680-76,963,676) | 10,594,825 (3,550,217-30,371,906) | 12,580,160 (4,264,337-31,142,915) |
| Out-of-pocket^a^ | 569,207 (213,179-1,112,470) | 103,647 (31,017-279,801) | 182,846 (60,585-450,155) |
| Indirect cost^a^ | 1,406,942 (494,547-3,129,303) | 370,614 (92,360-1,109,009) | 451,951 (140,550-1,266,255) |
| **Medically-attended mild illness (outpatient consultation)** | | | |
| **Total Cost ($)** | **111,189,136 (35,813,890-181,671,334)** | **73,868 (39,034-171,761)** | **292,157 (107,450-589,041)** |
| Direct Cost | 77,101,116 (29,612,000-105,681,673) | 51,221 (32,275-99,916) | 202,589 (88,843-342,656) |
| Healthcare system | 70,633,993 (27,751,433-95,255,523) | 46,925 (30,247-90,059) | 185,596 (83,261-308,851) |
| Out-of-pocket^a^ | 6,467,123 (1,860,567-10,426,150) | 4,296 (2,028-9,857) | 16,993 (5,582-33,805) |
| Indirect cost^a^ | 34,088,021 (6,201,890-75,989,661) | 22,646 (6,760-71,844) | 89,569 (18,607-246,385) |
| **Non-medically-attended mild and severe illness** | | | |
| **Total Cost ($)** | **114,199,113 (27,963,705-223,455,309)** | **429,831 (126,957-1,344,509)** | **841,769 (276,534-2,559,655)** |
| Direct Cost | 33,205,995 (13,496,244-44,319,798) | 58,625 (28,199-173,833) | 163,996 (74,374-434,139) |
| Healthcare system | Not applicable | Not applicable | Not applicable |
| Out-of-pocket^a^ | 33,205,995 (13,496,244-44,319,798) | 58,625 (28,199-173,833) | 163,996 (74,374-434,139) |
| Indirect cost^a^ | 80,993,118 (14,467,461-179,135,510) | 371,206 (98,758-1,170,675) | 677,773 (202,160-2,125,516) |
| **Total** | | | |
| **Total Cost ($)** | **266,526,964 (79,490,001-486,332,091)** | **11,572,784 (3,839,586-3,3276,984)** | **14,348,884 (4,849,456-36,008,021)** |
| Direct Cost | 150,038,883 (58,326,103-228,077,617) | 10,808,318 (3,641,708-30,925,456) | 13,129,591 (4,488,139-32,369,865) |
| Healthcare system | 109,796,558 (42,756,113-172,219,199) | 10,641,750 (3,580,464-30,461,965) | 12,765,756 (4,347,598-31,451,766) |
| Out-of-pocket^a^ | 40,242,325 (15,569,990-55,858,418) | 166,568 (61,244-463,491) | 363,835 (140,541-918,099) |
| Indirect cost^a^ | 116,488,081 (21,163,898-258,254,474) | 764,466 (197,878-2,351,528) | 1,219,293 (361,317-3,638,156) |

Abbreviations: CI: confidence intervals; PDE: patient per day equivalent.

^a^ Patient and caregiver.

**Table S3:** Estimated mean annual economic burden of influenza-associated influenza-like illness and severe acute respiratory illness using the World Health Organization toolkit with and without modifications in South Africa, 2013-2015 (main analysis using influenza-associated illness specific costs).

|  | **Toolkit with modifications^c^** | | **Toolkit without modifications** | |
| --- | --- | --- | --- | --- |
| **Parameter** | **Mean value per illness episode (95% CI)** | **Total**  **(95% CI)** | **Mean value per illness episode (95% CI)** | **Total**  **(95% CI)** |
| **Medically-attended influenza-associated severe acute respiratory illness** | | | | |
| **Total absenteeism (days)** | **3.3 (1.3-6.6)** | **86,158 (33,193-174,106)** | **3.3 (1.3-6.6)** | **86,158 (33,193-174,106)** |
| Absenteeism from school | 0.5 (0.1-1.0) | 12,273 (2,780-25,925) | 0.5 (0.1-1.0) | 12,273 (2,780-25,925) |
| Absenteeism from work^a^ | 2.8 (1.6-4) | 73,885 (42,853-104,917) | 2.8 (1.6-4) | 73,885 (42,853-104,917) |
| **Total Cost ($)** | **734 (421-1,135)** | **19,463,647 (6,354,207-44,901,083)** | **734 (421-1,135)** | **19,463,647 (6,354,207-44,901,083)** |
| Direct Cost | 700 (402-1,080) | 18,547,120 (6,069,159-42,725,743) | 700 (402-1,080) | 18,547,120 (6,069,159-42,725,743) |
| Healthcare system | 686 (394-1,060) | 18,176,321 (5,946,286-41,952,408) | 686 (394-1,060) | 18,176,321 (5,946,286-41,952,408) |
| Out-of-pocket^a^ | 14 (8-20) | 370,799 (122,873-773,335) | 14 (8-20) | 370,799 (122,873-773,335) |
| Indirect cost^a^ | 34 (19-55) | 916,526 (285,048-2,175,340) | 34 (19-55) | 916,526 (285,048-2,175,340) |
| **Medically-attended influenza-associated influenza-like illness** | | | | |
| **Total absenteeism (days)** | **1.4 (0.6-2.5)** | **1,269,516 (532,558-2,315,169)** | **1.4 (0.6-2.5)** | **1,269,516 (532,558-2,315,169)** |
| Absenteeism from school | 0.9 (0.5-1.5) | 869,832 (446,980-1,358,291) | 0.9 (0.5-1.5) | 869,832 (446,980-1,358,291) |
| Absenteeism from work^a^ | 0.4 (0.3-0.6) | 399,684 (287,772-511,596) | 0.4 (0.3-0.6) | 399,684 (287,772-511,596) |
| **Total Cost ($)** | **40 (28-54)** | **36,632,957 (21,487,850-5,9146,652)** | **40 (28-54)** | **36,632,957 (21,487,850-5,9146,652)** |
| Direct Cost | 27 (23-30) | 25,031,105 (17,692,691-33,233,301) | 27 (23-30) | 25,031,105 (17,692,691-33,233,301) |
| Healthcare system | 25 (22-27) | 22,830,021 (16,554,144-29,677,863) | 25 (22-27) | 22,830,021 (16,554,144-29,677,863) |
| Out-of-pocket^a^ | 2 (1-3) | 2,201,084 (1,138,547-3,555,438) | 2 (1-3) | 2,201,084 (1,138,547-3,555,438) |
| Indirect cost^a^ | 13 (5-24) | 11,601,852 (3,795,158-25,913,351) | 13 (5-24) | 11,601,852 (3,795,158-25,913,351) |
| **Non-medically-attended influenza-associated influenza-like illness** | | | | |
| **Total absenteeism (days)** | **1.3 (0.6-2.6)** | **3,164,904 (1,527,851-6,225,689)** | **0.8 (0.4-1.3)** | **1,860,175 (928,289-3,061,321)** |
| Absenteeism from school | 0.6 (0.3-0.9) | 1,462,644 (837,082-2,246,283) | 0.6 (0.4-0.9) | 1,457,473 (835,911-2,235,359) |
| Absenteeism from work^b^ | 0.7 (0.3-1.7) | 1,702,260 (690,769-3,979,406) | 0.2 (0.1-0.3) | 402,702 (92,378-825,962) |
| **Total Cost ($)** | **17 (7-35)** | **39,203,410 (17,587,008-82,203,095)** | **13 (8-20)** | **31,544,030 (15,196,547-57,457,355)** |
| Direct Cost | 5 (3-6) | 11,359,950 (8,250,426-15,268,105) | 5 (3-6) | 11,220,360 (8,209,629-14,933,850) |
| Healthcare system |  | Not applicable | Not applicable | Not applicable |
| Out-of-pocket^b^ | 5 (3-6) | 11,359,950 (8,250,426-15,268,105) | 5 (3-6) | 11,220,360 (8,209,629-14,933,850) |
| Indirect cost^b^ | 12 (4-28) | 27,843,460 (9,336,582-66,934,990) | 9 (3-18) | 20,323,670 (6,986,918-42,523,505) |
| **Total** | | | | |
| **Total absenteeism (days)** | **1.4 (0.6-2.6)** | **4,520,578 (2,093,602-8,714,964)** | **1.0 (0.5-1.7)** | **3,215,849 (1,494,040-5,550,596)** |
| Absenteeism from school | 0.7 (0.4-1.1) | 2,344,749 (1,286,842-3,630,499) | 0.7 (0.4-1.1) | 2,339,578 (1,285,671-3,619,575) |
| Absenteeism from work^a^ | 0.7 (0.3-1.4) | 2,175,829 (1,021,394-4,595,919) | 0.3 (0.1-0.4) | 876,271 (423,003-144,2475) |
| **Total Cost ($)** | **29 (14-56)** | **95,300,013 (45,429,064-186,250,830)** | **26 (15-41)** | **87,640,633 (43,038,603-161,505,090)** |
| Direct Cost | 17 (10-27) | 54,938,175 (32,012,276-91,227,149) | 16 (11-23) | 54,798,585 (31,971,479-90,892,894) |
| Healthcare system | 12 (7-22) | 41,006,342 (22,500,430-71,630,271) | 12 (8-18) | 41,006,342 (22,500,430-71,630,271) |
| Out-of-pocket^a^ | 4 (3-6) | 13,931,833 (9,511,846-19,596,878) | 4 (3-5) | 13,792,243 (9,471,049-19,262,623) |
| Indirect cost^a^ | 12 (4-29) | 40,361,838 (13,416,788-95,023,681) | 10 (4-18) | 32,842,048 (11,067,124-70,612,196) |

Abbreviations: CI: confidence intervals.

^a^ Patient and caregiver.

^b^ Patient and caregiver for the analysis using the toolkit with modifications and patient only for the analysis using the toolkit with modifications.

^c^ Modifications included: (i) addition of direct and indirect cost for patient and caregiver also for non-medically attended severe illness, and (ii) addition of indirect costs for patient and caregiver also for non-medically-attended mild illness.

**Table S4:** Estimated mean annual economic burden of influenza-associated influenza-like illness and severe acute respiratory illness using the World Health Organization toolkit with and without modification in South Africa, 2013-2015 (sensitivity analysis using PDE and overall consultation costs).

|  | **Toolkit with modifications^c^** | | **Toolkit without modifications** | |
| --- | --- | --- | --- | --- |
| **Parameter** | **Mean value per illness episode (95% CI)** | **Total**  **(95% CI)** | **Mean value per illness episode (95% CI)** | **Total**  **(95% CI)** |
| **Medically-attended influenza-associated severe acute respiratory illness** | | | | |
| **Total Cost ($)** | **1,011 (600-1,427)** | **26,799,059 (9,056,365-56,450,094)** | **1,011 (600-1,427)** | **26,799,059 (9,056,365-56,450,094)** |
| Direct Cost | 976 (581-1,372) | 25,882,532 (8,771,317-54,274,754) | 976 (581-1,372) | 25,882,532 (8,771,317-5,427,4754) |
| Healthcare system | 962 (573-1,352) | 25,511,733 (8,648,444-5,3501,419) | 962 (573-1,352) | 25,511,733 (8,648,444-5,3501,419) |
| Out-of-pocket^a^ | 14 (8-20) | 370,799 (122,873-773,335) | 14 (8-20) | 370,799 (122,873-773,335) |
| Indirect cost^a^ | 35 (19-55) | 916,526 (285,048-2,175,340) | 35 (19-55) | 916,526 (285,048-2,175,340) |
| **Medically-attended influenza-associated influenza-like illness** | | | | |
| **Total Cost ($)** | **41 (29-56)** | **37,843,203 (21,915,799-61,952,022)** | **41 (29-56)** | **37,843,203 (21,915,799-61,952,022)** |
| Direct Cost | 28 (24-33) | 26,241,351 (18,120,640-36,038,671) | 28 (24-33) | 26,241,351 (18,120,640-36,038,671) |
| Healthcare system | 26 (22-30) | 24,040,267 (16,982,093-32,483,233) | 26 (22-30) | 24,040,267 (16,982,093-32,483,233) |
| Out-of-pocket^a^ | 2 (1-3) | 2,201,084 (1,138,547-3,555,438) | 2 (1-3) | 2,201,084 (1,138,547-3,555,438) |
| Indirect cost^a^ | 13 (5-24) | 11,601,852 (3,795,158-25,913,351) | 13 (5-24) | 11,601,852 (3,795,158-25,913,351) |
| **Non-medically-attended influenza-associated influenza-like illness** | | | | |
| **Total Cost ($)** | **17 (7-35)** | **39,203,410 (17,587,008-82,203,095)** | **13 (8-20)** | **31,544,030 (15,196,547-57,457,355)** |
| Direct Cost | 5 (3-6) | 11,359,950 (8,250,426-15,268,105) | 5 (3-6) | 11,220,360 (8,209,629-14,933,850) |
| Healthcare system |  | Not applicable | Not applicable | Not applicable |
| Out-of-pocket^b^ | 5 (3-6) | 11,359,950 (8,250,426-15,268,105) | 5 (3-6) | 11,220,360 (8,209,629-14,933,850) |
| Indirect cost^b^ | 12 (4-28) | 27,843,460 (9,336,582-66,934,990) | 9 (3-18) | 20,323,670 (6,986,918-42,523,505) |
| **Total** | | | | |
| **Total Cost ($)** | **31 (15-60)** | **103,845,671 (48,559,171-200,605,211)** | **29 (16-45)** | **96,186,291 (46,168,710-175,859,471)** |
| Direct Cost | 19 (11-32) | 63,483,833 (35,142,383-105,581,530) | 19 (12-27) | 63,344,243 (35,101,586-105,247,275) |
| Healthcare system | 15 (8-26) | 49,552,000 (25,630,537-85,984,652) | 15 (9-22) | 49,552,000 (25,630,537-85,984,652) |
| Out-of-pocket^a^ | 4 (3-6) | 13,931,833 (9,511,846-19,596,878) | 4 (3-5) | 13,792,243 (9,471,049-19,262,623) |
| Indirect cost^a^ | 12 (4-29) | 40,361,838 (13,416,788-95,023,681) | 10 (4-18) | 32,842,048 (11,067,124-70,612,196) |

Abbreviations: CI: confidence intervals; PDE: patient per day equivalent.

^a^ Patient and caregiver.

^b^ Patient and caregiver for the analysis using the toolkit with modifications and patient only for the analysis using the toolkit with modifications.

^c^ Modifications included: (i) addition of direct and indirect cost for patient and caregiver also for non-medically attended severe illness, and (ii) addition of indirect costs for patient and caregiver also for non-medically-attended mild illness.

**Table S5:** Estimated mean annual years of life lost (YLL) from influenza-associated severe acute respiratory illness death in South Africa, 2013-2015.

| **Age (in years)** | **YLL (95% CI)** |
| --- | --- |
| <1 | 16,205 (10,762-22,823) |
| 1-4 | 6,926 (4,533-10,200) |
| 5-19 | 4,500 (1,262-9,055) |
| 20-44 | 21,116 (12,951-32,063) |
| 45-64 | 10,290 (5,082-16,065) |
| ≥65 | 6,660 (1,404-13,722) |
| <5 | 23,131 (15,295-33,023) |
| ≥5 | 42,566 (20,699-70,905) |
| All | 65,697 (35,994-103,928) |

**REFERENCES**

1. Statistics South Africa. Mid-year population estimates: 2013. Available at: <https://www.statssa.gov.za/publications/P0302/P03022013.pdf>. Accessed on 18 Jan 2018. [↑](#endnote-ref-1)
2. Statistics South Africa. Mid-year population estimates: 2014. Available at: <https://www.statssa.gov.za/publications/P0302/P03022014.pdf>. Accessed on 18 Jan 2018. [↑](#endnote-ref-2)
3. Statistics South Africa. Mid-year population estimates: 2015. Available at: <https://www.statssa.gov.za/publications/P0302/P03022015.pdf>. Accessed on 18 Jan 2018. [↑](#endnote-ref-3)
4. Tempia S, Walaza S, Moyes J, et al. Quantifying how different clinical presentations, levels of severity and healthcare attendance shape the burden of influenza-associated illness: a modelling study from South Africa. Clin Infect Dis. **2018**. doi: 10.1093/cid/ciy1017 . [↑](#endnote-ref-4)
5. Tempia S, Walaza S, Moyes J, et al. [The Effects of the Attributable Fraction and the Duration of Symptoms on Burden Estimates of Influenza-Associated Respiratory Illnesses in a High HIV-Prevalence Setting, South Africa, 2013-2015.](https://www.ncbi.nlm.nih.gov/pubmed/29210203) Influenza Other Respir Viruses. **2018**;12(3):360-373. [↑](#endnote-ref-5)
6. Cohen C, Moyes J, Tempia S, et al. Severe influenza-associated lower respiratory tract infection in a high HIV-Prevalence setting – South Africa, 2009-2011. Emerg Infec Dis. **2013**; 19(11):1766-74. [↑](#endnote-ref-6)
7. Pretorius MA, Madhi SA, Cohen C, et al. Respiratory viral coinfections identified by a 10-plex real-time reverse-transcription polymerase chain reaction assay in patients hospitalized with severe acute respiratory illness - South Africa, 2009-2010. J Infcet Dis. **2012**; 206(S1):S159-65. [↑](#endnote-ref-7)
8. Kyeyagalire R, Tempia S, Cohen AL, et al. [Hospitalizations associated with influenza and respiratory syncytial virus among patients attending a network of private hospitals in South Africa, 2007-2012.](https://www.ncbi.nlm.nih.gov/pubmed/25510622) BMC Infect Dis. **2014**;14:694. [↑](#endnote-ref-8)
9. National Department of Health. Uniform Patient Fee Schedule. <http://www.health.gov.za/index.php/shortcodes/2015-03-29-10-42-47/2015-04-30-09-10-23/uniform-patient-fee-schedule/category/448-upfs-2018>. Accessed on 21 November 2018. [↑](#endnote-ref-9)
10. National Department of Health. Single Exit Price Documents – Medicine Prices. <http://www.health.gov.za/index.php/single-exit-price-documents>. Accessed on 21 November 2018. [↑](#endnote-ref-10)
11. National Department of Health. National Health Act, Regulations, Reference Price List. <https://www.gov.za/sites/default/files/31469_1236.pdf>. Accessed on 18 Jan 2018. [↑](#endnote-ref-11)
12. National Health Laboratory Service. State Price List. <https://www.google.com/url?sa=t&rct=j&q=&esrc=s&source=web&cd=1&cad=rja&uact=8&ved=2ahUKEwiOu6y7qOXeAhXlCsAKHaclC7cQFjAAegQICRAC&url=http%3A%2F%2Fwww.health.gov.za%2Findex.php%2Fshortcodes%2F2015-03-29-10-42-47%2F2015-04-30-09-10-23%2Funiform-patient-fee-schedule%2Fcategory%2F221-u2014%3Fdownload%3D895%3Anhls-state-price-list-2013-annexure-m&usg=AOvVaw1-pJVdlBG3sX8vf0DOk3F6>. Accessed on 21 November 2018. [↑](#endnote-ref-12)
13. Health System Trust. District Health Barometer: 2013/2014. Available at: <http://www.hst.org.za/publications/District%20Health%20Barometers/DHB_2013_14_web.pdf>. Accessed on 18 Jan 2018. [↑](#endnote-ref-13)
14. Health System Trust. District Health Barometer: 2014/2015. Available at: <http://www.hst.org.za/publications/District%20Health%20Barometers/Complete_DHB_2014_15_linked.pdf>. Accessed on 18 Jan 2018. [↑](#endnote-ref-14)
15. Greenland K, Schmidt WP. Household cost of illness: Summary of Findings from Four Countries. Available at: <https://blogs.lshtm.ac.uk/envhealthgroup/files/2015/05/Household-Cost-of-Illness-Summary-of-findings-from-four-countries.pdf>. Accessed on 18 January 2018. [↑](#endnote-ref-15)
16. Wong KK, von Mollendorf C, Martinson NA, et al. Healthcare utilization for common infectious diseases syndromes in Soweto and Klerksdorp, South Africa. Pan Afr Med J. **2018**; in press. [↑](#endnote-ref-16)
17. McAnerney JM, Cohen C, Cohen AL, et al. Healthcare utilization patterns for common syndromes in Msunduzi Municipality, Pietermaritzburg, KwaZulu-Natal Province, South Africa, 2013. S Afr Med J. **2018**; in press. [↑](#endnote-ref-17)
18. South Africa National Treasury. A national minimum wage for South Africa, 2016. Available at: <http://www.treasury.gov.za/publications/other/NMW%20Report%20Draft%20CoP%20FINAL.PDF>. Accessed on 18 Jan 2018. [↑](#endnote-ref-18)
19. Statistic South Africa. Quarterly Labour Force Survey – Quarter 1: 2018. Available at: <http://www.statssa.gov.za/publications/P0211/P02111stQuarter2018.pdf>. Accessed on 18 January 2018. [↑](#endnote-ref-19)
20. World Health Organization. Life tables by country. Available at: <http://apps.who.int/gho/data/view.main.61540?lang=en>. Accessed on 18 Jan 2018. [↑](#endnote-ref-20)
